# Maternal Separation Early in Life Alters the Expression of Genes *Npas4* and *Nr1d1* in Adult Female Mice: Correlation with Social Behavior

**DOI:** 10.1155/2020/7830469

**Published:** 2020-03-03

**Authors:** Yuliya A. Ryabushkina, Vasiliy V. Reshetnikov, Natalya P. Bondar

**Affiliations:** ^1^Laboratory of Gene Expression Regulation, Institute of Cytology and Genetics, Siberian Branch of Russian Academy of Sciences (SB RAS), Prospekt Lavrentyeva 10, Novosibirsk, 630090, Russia; ^2^Novosibirsk State University, Pirogova Street, 2, Novosibirsk, 630090, Russia

## Abstract

Early-life stress affects neuronal plasticity of the brain regions participating in the implementation of social behavior. Our previous studies have shown that brief and prolonged separation of pups from their mothers leads to enhanced social behavior in adult female mice. The goal of the present study was to characterize the expression of genes (which are engaged in synaptic plasticity) *Egr1*, *Npas4*, *Arc*, and *Homer1* in the prefrontal cortex and dorsal hippocampus of adult female mice with a history of early-life stress. In addition, we evaluated the expression of stress-related genes: glucocorticoid and mineralocorticoid receptors (*Nr3c1* and *Nr3c2*) and *Nr1d1*, which encodes a transcription factor (also known as *REVERBα*) modulating sociability and anxiety-related behavior. C57Bl/6 mice were exposed to either maternal separation (MS, 3 h once a day) or handling (HD, 15 min once a day) on postnatal days 2 through 14. In adulthood, the behavior of female mice was analyzed by some behavioral tests, and on the day after the testing of social behavior, we measured the gene expression. We found increased *Npas4* expression only in the prefrontal cortex and higher *Nr1d1* expression in both the prefrontal cortex and dorsal hippocampus of adult female mice with a history of MS. The expression of the studied genes did not change in HD female mice. The expression of stress-related genes *Nr3c1* and *Nr3c2* was unaltered in both groups. We propose that the upregulation of *Npas4* and *Nr1d1* in females with a history of early-life stress and the corresponding enhancement of social behavior may be regarded as an adaptation mechanism reversing possible aberrations caused by early-life stress.

## 1. Introduction

A large body of evidence that has accumulated to date indicates that exposure to stressful events early in life affects subsequent development and predisposition to various psychiatric disorders [1, 2]. Furthermore, it has been extensively shown that consequences of early-life stress in mice are significantly sex biased. Although the influence on anxiety is reported to be roughly equal between the sexes, disturbances in memory formation and learning abilities are detected more frequently in males than in females, and this effect is more stable than the effect on females; however, the directionality is generally comparable between the sexes (see reviews [3, 4]).

The most contradictory effects of early-life stress are the effects on social behavior. Although most studies confirm disruption/downregulation of social contacts in males, according to various tests [5–8], the amount of research on females is too small to draw a general conclusion. Some authors have stated that maternal separation results in shorter time investigating a partner in adult [7, 9] or juvenile females [5, 10], while others suggest that maternal separation has no effect on female social behavior [11–13].

In our previous study [14], we investigated sex-specific changes in behavior under the influence of two types of stress: brief (15 min/day) and prolonged separation (3 h/day) of pups from their mothers. We found that both types of stress result in enhanced social behavior in female mice. On the other hand, there are reports of greater anxiety [14] and worsening of cognitive functions [15] in females with a history of prolonged maternal separation. Thus, similar enhancements of social behavior develop during different molecular changes in the brain, possibly indicating different mechanisms underlying the changes in social behavior after exposure to one of these two types of stress.

Normal development of social repertoire requires correct and precisely timed development of the brain regions related to social behavior. Overall, social behaviors involve and require a distributed set of neural networks including frontal and temporal cortices and limbic system structures, e.g., the nucleus accumbens, hypothalamus, and amygdala, which interact to produce social and emotional behaviors [16, 17]. The hippocampus is often included in this set of networks [18]. Of note, the ventral hippocampus is primarily involved in the regulation of emotional states and social behavior and is connected with the amygdala and hypothalamus. By contrast, the dorsal hippocampus mainly participates in cognitive functions and information processing and is connected with cortical areas [19]. Early-life stress negatively affects the formation of the brain regions taking part in the implementation of social behavior. For example, in the prefrontal cortex of rats with a history of early-life stress, the myelination level was found to be lower than that in control animals [20]. There is a report of a decrease in the volumes of the hippocampus in adult animals that were subjected to stress early in life [21, 22]. Early-life stress impairs both structural and functional plasticity within the brain regions important for the implementation of social behavior [23–26]. This impairment manifests itself as atrophy of the basal dendritic trees, lower spine density in layer II/III pyramidal neurons, and impairment of long-term potentiation processes in the medial prefrontal cortex [23, 25] and in the CA1 zone of the hippocampus [24]. Among groups of genes/proteins that are strongly involved in synaptic plasticity, there are immediate early genes [27], whose products partake in several distinct processes required for long-term synaptic changes and memory formation [28].

The specific aim of the present study was to characterize the expression of immediate early genes in the prefrontal cortex of adult female mice as a marker of modified neuroplasticity elicited both by stress early in life and by previous social interaction. In addition, we analyzed the expression of these genes in the dorsal hippocampus, as a region affected by stress early in life, as demonstrated by us previously [15]. In that study [15], by means of the same model of stress in females, we showed that prolonged maternal separation reduces the number of mature neurons in the CA3 area of the dorsal hippocampus and impairs long-term spatial and recognition memory. Thus, in the present study, we wanted to estimate how these observed behavioral changes in emotional states are related to the neuroplasticity of the dorsal hippocampus.

In this experiment, we compared groups of mice subjected to different types of stress: brief (15 min/day) and prolonged (3 h/day) maternal separation. One day after the testing of social behavior, we assessed the expression of some neuronal-plasticity–associated genes (*Egr1*, *Npas4*, *Arc*, and *Homer1*) in the dorsal hippocampus and prefrontal cortex of female mice. This choice of genes is based on the existing data on their involvement in social and emotional behavior. *Egr1* is expressed widely in different brain areas responsible for the control of cognition, emotional responses, and social behavior [29, 30]. *Npas4* regulates the excitatory–inhibitory balance and plays a key part in social behavior and cognition [31, 32]. *Arc* and *Homer1a* are involved in the maintenance of structural and functional modifications of dendrites that lead to long-term changes in synaptic efficacy in hippocampal and neocortical neuronal networks [33]. In addition, we assessed the expression of specific stress-responsive genes—glucocorticoid and mineralocorticoid receptors, *Nr3c1* and *Nr3c2* (which are important mediators of the stress response and interact with proteins that are activated by neuronal activity [34]) and *Nr1d1*, which encodes the transcription factor *REVERBα* modulating sociability and anxiety-related behavior [35].

## 2. Methods

### 2.1. Animals

C57BL/6J mice were housed at the Center for Genetic Resources of Laboratory Animals (RFMEFI62117X0015), the Institute of Cytology and Genetics (SB RAS, Novosibirsk, Russia). The animals were housed under standard conditions (12 : 12 h light/dark cycle, lights on at 8.00 a.m.; feed pellets and water were available *ad libitum*).

### 2.2. Maternal Separation

This procedure was described in another work [14]. Briefly, virgin males and females were used for mating. Pregnant females were individually housed with paper nesting material during their third week of gestation. Only litters containing 4–6 pups were employed in the experiments. The pups were subjected to brief or prolonged separation from their mothers from postnatal day 2 (PND2) to PND14 daily. In the brief maternal separation condition (handling (HD)), the pups were separated from their dams for 15 min once a day, whereas for the prolonged separation (maternal separation (MS)), the pups were separated for 180 min once a day. All the procedures were performed from 13:00 to 16:00 h in the light phase of the day. Nonhandled mice were not separated from dams (normal conditions, i.e., no-treatment control (NC)). The behavioral tests were conducted on PND85–PND110 in the following order: plus maze, open field, and the social interaction test (one test per day). Results of the detailed behavioral analysis were described previously [14]. In the current study, only C57BL/6 adult female mice with a history of early-life stress and control mice without any stressful experience were analyzed.

### 2.3. Tissue Collection

Animals were killed by decapitation between 10 and 12 o'clock the next day after the social interaction test (~PND 100). Brains were removed, the prefrontal cortex was dissected and snap-frozen in liquid nitrogen in 1.5 ml plastic tubes, and the rest of the brain was embedded in the Tissue-Tek O.C.T. Compound (Sakura Finetek U.S.A., Inc., USA). All the tissue samples were stored at −80°C before use. Trunk blood was collected, left at room temperature for 1 h, and then centrifuged at 3000 × *g* for 10 min. The resultant blood serum was stored at −80°C until analysis.

For the isolation of the dorsal hippocampus, frozen brains were cut into coronal slices with a cryostat, Microm HM 550. Two 150 *μ*m slices were prepared according to the Allen Brain Atlas (Bregma from −1.86 to −2.16, levels 73–76), and the dorsal hippocampus was isolated from slices by means of glass microsticks. Tissue punches from the right and left hemispheres were combined for RNA isolation.

### 2.4. RNA Extraction and Real-Time PCR

RNA was extracted from frozen tissue with PureZol (Bio-Rad, USA) in accordance with the manufacturer's protocol. The obtained samples of RNA were purified on Agencourt RNAClean XP beads (Beckman Coulter, Germany) and were diluted in double-distilled water. RNA quality and quantity were evaluated on a NanoDrop 2000 spectrophotometer.

Complementary DNA (cDNA) was synthesized using the kits produced by Syntol (Russia). The reaction included total RNA (1 *μ*g from the hippocampus or 0.5 *μ*g from the prefrontal cortex) and a mixture of random hexanucleotides as primers; all the procedures were carried out according to the manufacturer's protocols.

Gene expression was assessed by real-time PCR on a CFX96 Real-Time PCR Detection System (Bio-Rad, USA). We evaluated the expression of genes *Egr1*, *Npas4*, *Arc*, *Homer1*, *Nr3c1*, *Nr3c2*, and *Nr1d1*. For *Homer1*, we analyzed the expression of two isoforms: *Homer1a* and *Homer1b/c.* Each reaction was carried out in a mixture of cDNA, 0.25 mM dNTPs, 2.5 mM MgCl_2_, 10 mM each primer, 0.25 U of SynTaq DNA polymerase, and the buffer with EVA-Green (Syntol, Russia). Primers were designed with Primer-BLAST (NCBI; Supplementary [Supplementary-material supplementary-material-1]). The reaction parameters were as follows: 95°C for 5 min followed by 38 cycles at 95°C for 10 s and at 60°C for 20 s. After completion of the PCR, product specificity was assessed by an analysis of melting curves. Each reaction was run in triplicate. The amplification efficiency was 90% to 110% for each primer pair. The results of PCR were analyzed by the ΔΔCt method and normalized to the expression of *β*-actin (*Actb*) as a reference gene.

### 2.5. An Immunoassay of 17*β*-Estradiol

Serum 17*β*-estradiol was quantified in duplicate by means of the commercially available enzyme-linked immunosorbent assay (ELISA) kit (R&D Systems, KGE014) following the manufacturer's protocols.

### 2.6. Statistical Analysis

The normality of distribution and homogeneity of variances were tested by the Shapiro–Wilks test and Levene's test, respectively. The data were analyzed by one-way ANOVA (with the type of stress as a factor) and Fisher's least significant difference (LSD) test as a *post hoc* analysis. Differences between the groups were considered statistically significant at *p* < 0.05 and were assumed to show a tendency at *p* < 0.1. Associations between levels of gene expression, behavioral domains, and 17*β*-estradiol concentration were assessed by Pearson's correlation analysis. The statistical analyses were performed in Statistica 8.0 software.

## 3. Results

**Figure 1:**
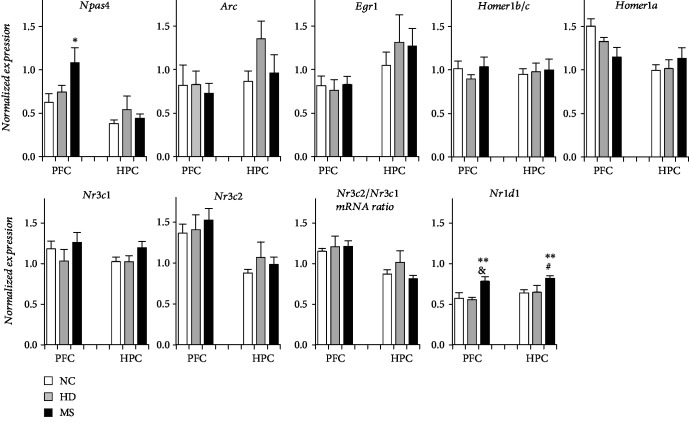
Effects of early-life stress on gene expression in the prefrontal cortex and dorsal hippocampus. Data are presented as mean ± SEM. HPC: hippocampus; PFC: prefrontal cortex; NC: no-treatment control; HD: handling, MS: maternal separation. ^∗^*p* < 0.05, ^∗∗^*p* < 0.01 as compared with the NC group; ^&^*p* < 0.1 (tendency), ^#^*p* < 0.05 as compared with the HD group (*post hoc* Fisher's LSD test). Numbers of mice in the groups: *n* = 11 in NC, *n* = 6 in HD, and *n* = 9 in MS.

We assessed the expression of four activity-regulated genes (*Egr1*, *Npas4*, *Arc*, and *Homer1*) in two brain regions: the dorsal hippocampus and prefrontal cortex (Figure 1). We found that only the *Npas4* mRNA level changed under the influence of early-life stress and only in one brain region: the prefrontal cortex (*F*(2, 22) = 3.65, *p* = 0.042). Fisher's LSD test revealed increased expression of cortical *Npas4* mRNA in MS females as compared to NC females (*p* = 0.014). Expression of the other activity-regulated genes did not change either in the dorsal hippocampus or in the prefrontal cortex. Levels of gene expression in the HD group did not differ from those in the NC group.

Early-life stress altered the *Nr1d1* mRNA level in the dorsal hippocampus and prefrontal cortex (*F*(2, 22) = 4.30, *p* = 0.026, and *F*(2, 22) = 4.89, *p* = 0.018). MS females featured an increase in both hippocampal and cortical levels of *Nr1d1* mRNA as compared to HD group females (*p* = 0.052 and *p* = 0.033) and to NC group females (*p* = 0.001 and *p* = 0.008).

Early-life stress did not alter the expression of stress-related genes *Nr3c1* and *Nr3c2* either in HD females or MS females in comparison with NC females.

**Figure 2:**
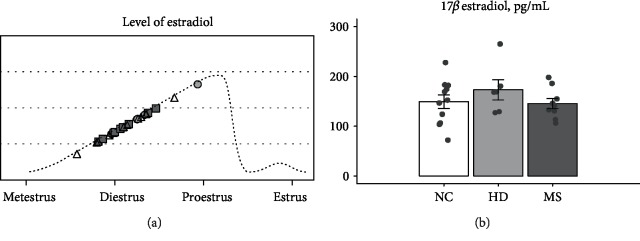
The effect of stress on the level of estradiol. (a) Schematic representation of estradiol dynamics during the estrous cycle of mice. Symbols indicate estradiol levels in the groups of experimental animals. Triangles: no-treatment control group (NC), circles: HD group, and squares: MS group. (b) The impact of early-life stress on the concentration of 17*β*-estradiol in the serum of female mice. Data are means ± SEM with *n* = 11 in the NC group, *n* = 6 in the HD group, and *n* = 9 in the MS group.

Because in females, the expression of some genes may depend on changes in sex hormones' levels, we determined the 17*β*-estradiol concentration in the blood serum of female mice. The distribution of 17*β*-estradiol levels among all the samples indicated that most of the females were in the diestrus or proestrus stage of the ovarian cycle (Figure 2). Only one NC mouse showed a low level of estradiol corresponding to the metestrus phase. We did not find any significant differences among the groups in estradiol levels. Correlation analysis revealed a significant association between the estradiol level and gene expression only for *Nr3c2* mRNA in the prefrontal cortex (*r*^2^ = 0.20, *p* = 0.027; [Supplementary-material supplementary-material-1]). For most genes, we did not detect a significant correlation between estradiol concentration and gene expression; this finding allows us to compare gene expression levels without considering the stage of the cycle.

**Figure 3:**
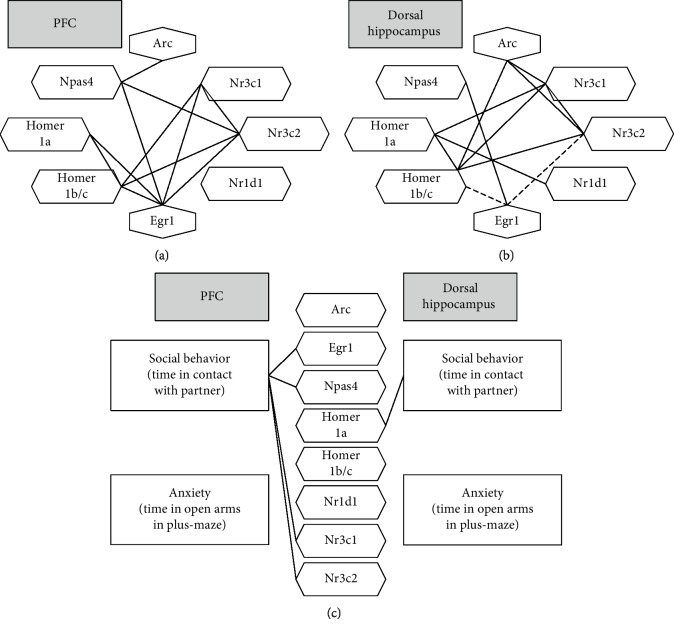
Correlations among expression levels of genes in the (a) prefrontal cortex and (b) dorsal hippocampus of female mice. (c) Correlations among gene expression levels and behavioral domains of social behavior and anxiety (behavioral data from [14]). Solid lines: positive correlations; dotted lines: negative correlations; *p* < 0.05 according to Pearson's correlation analysis.

We conducted the analysis of correlation among expression levels of the genes in the prefrontal cortex and hippocampus. Combined data of all the groups were subjected to Pearson's correlation analysis (Figures 3(a) and 3(b)). Correlation coefficients are presented in Supplementary [Supplementary-material supplementary-material-1]. We found significant correlations among several activity-regulated genes reflecting concordant changes in their expression under stress. mRNA expression of *Nr1d1* mostly did not correlate with that of the other genes, indicating its independent regulation.

To identify a possible link between a change in the expression of genes and the animals' psycho-emotional state, we performed a correlation analysis between genes' expression levels and the parameters of anxious and social behavior that have been evaluated previously in the same animals [14]. We found a correlation of gene expression with the time spent in the open arms (elevated plus maze test) as an indicator of anxiety and with the time spent in contact with an unfamiliar partner (social interaction test) as an indicator of sociability. We found that expression of four of the eight studied genes in the prefrontal cortex and of one gene in the hippocampus positively correlated with the level of social behavior (Figure 3(c)). It is worth mentioning that these results may be regarded as indirect confirmation that gene expression in the prefrontal cortex is more strongly associated with social behavior than gene expression in the dorsal hippocampus is. We did not note any correlations between anxiety behavior and expression of the genes (correlation coefficients are presented in Supplementary [Supplementary-material supplementary-material-1]).

## 4. Discussion

In this work, we showed that brief and prolonged maternal separation early in life has a delayed effect on the expression of genes in the prefrontal cortex and dorsal hippocampus of adult female mice. This stress had the strongest influence on the expression of *Npas4* and *Nr1d1*.

### 4.1. Maternal Separation Leads to Higher *Npas4* Expression in the Prefrontal Cortex of Adult Females

The early-response transcription factor *Npas4* is one of the key regulators of the excitatory-inhibitory balance within neural circuits [32] and is expressed in neurons mainly in the frontal cortex and hippocampus [36, 37]. Usually, enhancement of *Npas4* expression occurs within the first hour after application of a stimulus; for instance, its expression in the hippocampus and frontal cortex increases in response to a social encounter, novelty forced swim stress, or foot shock [31, 38–40]. This expression enhancement is often accompanied by higher expression of other immediate early genes such as *Arc*, *Egr1*, and *c-Foc* [38, 39], and usually, their expression subsides within 4–5 h after the stimulus [40]. Nevertheless, it has been reported that chronic stress can additionally result in a prolonged alteration in the expression of *Npas4* and some other immediate early genes; for example, after lengthy social isolation, *Npas4* expression in the hippocampus remains low for at least a month [41].

In our study, we revealed that in adult MS females, *Npas4* expression was higher in the prefrontal cortex. The other genes under study did not significantly change their expression. It is known that as a transcription factor, Npas4 exerts control over the transcription of many other genes (e.g., *Arc*, *Egr1*, and *Bdnf*) [32, 36, 42]. Furthermore, in spite of the absence of significant changes in expression, the strong correlations observed among the expression levels of these genes in the prefrontal cortex suggest that immediate early genes—*Npas4*, *Arc*, *Egr1*, and *Homer1a*—show a concordant pattern of expression under the influence of external stimuli. In the dorsal hippocampus, we did not detect either changes in the expression of early response genes or correlations among their expression levels, suggesting that the observed changes are specific to the prefrontal cortex.

In this work, we evaluated the expression of genes in a day after the last behavioral test (social interaction test); therefore, the expression changes may be related specifically to neuronal activation as a consequence of social contacts among the mice. The analysis of correlation between behavioral parameters in various tests and expression levels of the studied genes indicated that the cortical expression of *Npas4*, *Arc*, and *Egr1* positively correlates specifically with this parameter, which reflects the level of social behavior but not the anxiety level. Even though enhanced social behavior (as compared with the control group) was uncovered both in the MS group and HD group, significant upregulation of *Npas4* was registered only in the MS group. Consequently, the observed alterations are possibly associated with a change in the basal level of gene expression under the influence of early-life stress or with a change in the degree of its activation after social contacts with a partner. It is possible that in the MS group, the upregulation of *Npas4* after behavioral testing is reversed more slowly than that in the control animals and in the HD group.

In another study, an increase in *Npas4* expression was detected in the hippocampus of mice subjected to prolonged exposure to an enriched environment early in life [42], and this enriched environment often also caused an enhancement of social behavior in the adult animals [43, 44]. Rats with higher hippocampal expression of *Npas4* [38] manifest enhanced exploratory behavior. On the contrary, *Npas4* knockout mice are hyperactive in a novel environment and are less socially active [31]. These observations support our hypothesis that *Npas4* overexpression is related to enhanced social behavior. Conversely, long-lasting exposure to a stressor such as chronic restraint [45], social isolation of juveniles [41], and chronic mild stress [46] lead to both weaker social behavior and lower hippocampal expression of *Npas4*. Accordingly, we believe that the upregulation of *Npas4* in the prefrontal cortex of females with a history of early-life stress—as well as the corresponding enhancement of social behavior—can be regarded as an adaptation mechanism that reverses the possible disturbances caused by early-life stress. *Npas4* and some other genes have been reported to have a neuroprotective effect, in particular, *Npas4* upregulation promotes the survival of hippocampal neurons in response to synaptic NMDA stimulation [47].

Thus, our findings suggest that the behavioral alterations in female mice with a history of MS (i.e., greater anxiety and enhancement of social behavior) may be associated with the level of *Npas4* expression and subsequent changes in the expression of its target genes. Nonetheless, the exact mechanism by which Npas4 influences social behavior requires further research because Npas4 regulates a large number of genes.

### 4.2. Early-Life Stress Increases the Expression of *Nr1d1* but Does Not Affect the Expression of Stress Response Genes

In this work, we demonstrated that the expression of a nuclear receptor gene, *Nr1d1* (which encodes the transcription factor also known as *REVERBα*), increases both in the dorsal hippocampus and in the prefrontal cortex of female mice with a history of MS. *Nr1d1* is a nuclear receptor that modulates gene transcription, and its function in circadian rhythm regulation has been studied most extensively. *Nr1d1* takes part in accessory loop regulation of clock genes' expression and in feedback to inhibit the CLOCK–BMAL1 heterodimer transactivation function. Several studies suggest that *Nr1d1* may play a role in reward-related processes [48, 49], mood-related behavior [35, 50, 51], and disorders associated with social deficits [52]. Upregulation of *Nr1d1* in medial prefrontal cortices has been detected during the formation of depression-like behavior in mice [53]. A recent human postmortem study indicates that clock genes (including *NR1D1*) are rhythmically expressed in the brain regions involved in mood regulation, but these rhythms are attenuated in subjects with major depressive disorder [54]. Suicide completers with or without a history of child abuse have a distinct pattern of DNA methylation of the genes implicated in neuronal plasticity including *NR1D1*. DNA methylation in the *NR1D1* promoter is greater in humans with a history of child abuse [55]. In our study of delayed effects of early-life stress on the genomic landscape of H3K4me3 in adult male mice [56], there was a significant increase in the amount of active-chromatin modification H3K4me3 in the promoter region of *Nr1d1*. These data are suggestive of a more active promoter of this gene in the animals with a history of MS. These results are well consistent with the upregulation of *Nr1d1* in MS females observed in the present study.

Circadian-rhythm amplitude is important for proper mood regulation. Recent evidence [50, 57] from *Nr1d1*-deficient mice reveals participation of *Nr1d1* in the modulation of midbrain and hippocampal dopamine activity due to regulation of tyrosine hydroxylase: the rate-limiting enzyme in dopamine production. *Nr1d1*'s actions in the dopaminergic system profoundly influence mood-related and emotional behaviors in mice. For this reason, it is possible that the enhancement of social behavior in MS females observed here is also related to an alteration of dopaminergic activity in the limbic system.

Consequently, enhanced expression of *Nr1d1* in the frontal cortex and dorsal hippocampus may influence the activity of clock genes and of dopamine, thereby modulating emotion and social behavior. Probably, this change in *Nr1d1* expression in MS females and the related possible aberration of circadian rhythms may explain greater susceptibility to psychiatric disorders at an adult age.

The circadian clock and the stress response system are closely connected [58]. Many clock gene promoters contain glucocorticoid response elements, and glucocorticoids synchronize peripheral and central circadian oscillators. The *Nr1d1* promoter contains several binding sites for glucocorticoid receptor (GR), and it has been demonstrated that GR activation can downregulate *Nr1d1* [59]. In turn, the t*ranscription factor* encoded by the *Nr1d1* gene regulates GR expression both by binding to its promoter and via the regulation of activity of the CLOCK–BMAL1 complex [60]. Nonetheless, in our experiment, the higher *Nr1d1* expression did not result in alteration of *Nr3c1* or *Nr3c2* expression in the prefrontal cortex and hippocampus of adult female mice. Similarly, in our recent study [61] on males, we did not detect changes in the expression of either *Nr3c1* or *Nr3c2* under the influence of early-life stress, in agreement with the absence of such changes in females, but prolonged maternal separation resulted in a higher *Nr3c2*/*Nr3c1* mRNA ratio in the hippocampus and hypothalamus in males. Thus, we did not detect an influence of early-life stress on *Nr3c1* or *Nr3c2* expression in females, at least under unstressful conditions. A substantial influence of early-life stress on the expression of hypothalamic–pituitary–adrenal axis–related genes is mostly seen in rat studies: early handling (brief separation) is associated with long-term overexpression of hippocampal GR and an attenuated stress response [62–64]. Conversely, prolonged maternal separation is reported to produce a vulnerable phenotype, with a decrease in the GR level and a prolonged neuroendocrine response to stress [62, 65]. In addition, maternal separation in rats strongly affects the adrenocortical functionality, by regulating GR expression in the dorsal CA1 [66]. In the research on early-life stress in mice, long-term changes in *Nr3c1* are *Nr3c2* expression are usually not found in either males or females [11, 67, 68], although there are isolated reports about enhanced hippocampal *Nr3c1* expression in adult MS and HD male mice [69] or decreased cortical *Nr3c1* expression in adult MS male mice [70]. Therefore, our data support the hypothesis that mice (in contrast to rats) are more resistant to the effects of early-life stress, at least at the level of regulation of the hypothalamic–pituitary–adrenal axis [11, 69].

## 5. Conclusion

In this study, we investigated the impact of brief and prolonged maternal separation on the expression of *Nr1d1* and of the early response genes whose protein products participate in neuronal plasticity and regulation of social behavior. Early-life experience can shape the developing brain and encode subsequent behavior of the individuals. Our previous studies have revealed that early-life experience leads to enhanced social behavior of females with a history of brief or prolonged maternal separation [14], and these alterations may affect the next generation too: the level of social behavior was also high (at least among males), and the social coping strategy was different in the descendants that were brought up by the mothers with a history of prolonged maternal separation [15, 71]. We can theorize that the upregulation of *Npas4* and *Nr1d1* in females with a history of early-life stress and the corresponding enhancement of social behavior may be considered an adaptation mechanism reversing the possible aberrations caused by early-life stress.

## Data Availability

The data used to support the findings of this study are available from the corresponding author upon request.

## Supplementary Materials

Supplementary Materials Table S1: sets of primers. Table S2: pairwise correlations of expression among the studied genes in the prefrontal cortex and dorsal hippocampus. Table S3: correlations between gene expression levels and parameters of anxious and social behavior. Figure S1: correlation between the relative mRNA level of genes and serum 17*β*-estradiol concentration.
