# Supplementary material for: Maternal Separation Early in Life Alters the Expression of Genes Npas4 and Nr1d1 in Adult Female Mice: Correlation with Social Behavior
Source: Behav Neurol. 2020 Mar 3;2020:7830469. doi: 10.1155/2020/7830469 (PMC7072106; doi:10.1155/2020/7830469)
Supplement: Supplementary Materials — Table S1: sets of primers. Table S2: pairwise correlations of expression among the studied genes in the prefrontal cortex and dorsal hippocampus. Table S3: correlations between gene expression levels and parameters of anxious and social behavior. Figure S1: correlation between the relative mRNA level of genes and serum 17β-estradiol concentration. [file 7830469.f1.docx]

**Table S1.** Sets of primers

| **Gene name** | **Forward 5′→3′** | **Reverse 5′→3′** | **Product size, bp** |
| --- | --- | --- | --- |
| *Arc* | CCTACAGAGCCAGGAGAATGAC | GAAGAGAGGATGGTGCTGGTG | **110** |
| *Egr1* | TTCAATCCTCAAGGGGAGCC | GAGAAGCGGCCAGTATAGGT | **158** |
| *Homer1a* | CAAACACTGTTTATGGACTG | TGCTGAATTGAATGTGTACC | **302** |
| *Homer1b,c* | TCACTGAGCTGGAGTGTGTTAG | GGTTACTGCGGAAAGCCTCT | **291** |
| *Npas4* | CTATACTCAGAAGGTCCAGAAGG | TCAGAGAATGAGGGTAGCACAGC | **152** |
| *Nr1d1* | CCAACAGTCTACGGCAAGGC | CGCACCATCAGCACCTCAAA | **241** |
| *Nr3c1* | ATGTATGACCAATGTAAACACA | GCTCTTCAGACCTTCCTTAG | **132** |
| *Nr3c2* | GTGTGTGGAGATGAGGC | GGACAGTTCTTTCTCCGAAT | **155** |
| *Actb* | CAATGAGCTGCGTGTGGC | GTACATGGCTGGGGTGTTGA | **127** |

**Table S2.** Pairwise correlations of expression among the studied genes in the prefrontal cortex and dorsal hippocampus

| **Prefrontal cortex** |  |  |  |  |  |  |  |  |  |
| --- | --- | --- | --- | --- | --- | --- | --- | --- | --- |
|  |  | ***Arc*** | ***Egr1*** | ***Homer1b/c*** | ***Homer1a*** | ***Npas4*** | ***Nr3c1*** | ***Nr3c2*** | ***Nr1d1*** |
| ***Arc*** | **r** | 1 | 0.1928 | -0.1193 | 0.1235 | 0.4807 | -0.2226 | -0.0064 | 0.026 |
|  | **p** |  | 0.356 | 0.570 | 0.557 | 0.015 | 0.285 | 0.976 | 0.902 |
| ***Egr1*** | **r** |  | 1 | 0.7285 | 0.7221 | 0.4306 | 0.5504 | 0.5066 | 0.2011 |
|  | **p** |  |  | 0.000 | 0.000 | 0.032 | 0.004 | 0.010 | 0.335 |
| ***Homer1b/c*** | **r** |  |  | 1 | 0.4758 | 0.3332 | 0.8338 | 0.7887 | 0.1522 |
|  | **p** |  |  |  | 0.016 | 0.104 | 0.000 | 0.000 | 0.468 |
| ***Homer1a*** | **r** |  |  |  | 1 | 0.1604 | 0.3099 | 0.2933 | 0.3002 |
|  | **p** |  |  |  |  | 0.444 | 0.132 | 0.155 | 0.145 |
| ***Npas4*** | **r** |  |  |  |  | 1 | 0.2494 | 0.4052 | 0.1095 |
|  | **p** |  |  |  |  |  | 0.229 | 0.045 | 0.602 |
| ***Nr3c1*** | **r** |  |  |  |  |  | 1 | 0.8292 | 0.2063 |
|  | **p** |  |  |  |  |  |  | 0.000 | 0.322 |
| ***Nr3c2*** | **r** |  |  |  |  |  |  | 1 | 0.301 |
|  | **p** |  |  |  |  |  |  |  | 0.144 |
| ***Nr1d1*** | **r** |  |  |  |  |  |  |  | 1 |
|  | **p** |  |  |  |  |  |  |  |  |
| **Dorsal hippocampus** |  |  |  |  |  |  |  |  |  |
|  |  | ***Arc*** | ***Egr1*** | ***Homer1b/c*** | ***Homer1a*** | ***Npas4*** | ***Nr3c1*** | ***Nr3c2*** | ***Nr1d1*** |
| ***Arc*** | **r** | 1 | -0.3188 | 0.5042 | 0.1594 | 0.2054 | 0.5073 | 0.6263 | -0.1444 |
|  | **p** |  | 0.138 | 0.014 | 0.468 | 0.347 | 0.013 | 0.001 | 0.511 |
| ***Egr1*** | **r** |  | 1 | -0.4769 | 0.0316 | 0.6084 | -0.2481 | -0.6084 | 0.0692 |
|  | **p** |  |  | 0.021 | 0.886 | 0.002 | 0.254 | 0.002 | 0.754 |
| ***Homer1b/c*** | **r** |  |  | 1 | 0.4795 | 0.0243 | 0.6911 | 0.6021 | -0.0612 |
|  | **p** |  |  |  | 0.021 | 0.912 | 0.000 | 0.000 | 0.781 |
| ***Homer1a*** | **r** |  |  |  | 1 | -0.0333 | 0.6082 | 0.2538 | 0.4653 |
|  | **p** |  |  |  |  | 0.880 | 0.002 | 0.243 | 0.025 |
| ***Npas4*** | **r** |  |  |  |  | 1 | -0.1126 | -0.3901 | -0.2588 |
|  | **p** |  |  |  |  |  | 0.609 | 0.066 | 0.233 |
| ***Nr3c1*** | **r** |  |  |  |  |  | 1 | 0.6213 | 0.2311 |
|  | **p** |  |  |  |  |  |  | 0.000 | 0.289 |
| ***Nr3c2*** | **r** |  |  |  |  |  |  | 1 | 0.0799 |
|  | **p** |  |  |  |  |  |  |  | 0.717 |
| ***Nr1d1*** | **r** |  |  |  |  |  |  |  | 1 |
|  | **p** |  |  |  |  |  |  |  |  |

**r**: Pearson’s correlation coefficient, **p**: p value.

**Table S3**. Correlations between gene expression levels and parameters of anxious and social behavior

|  |  | ***Arc*** | ***Egr1*** | ***Homer1b/c*** | ***Homer1a*** | ***Npas4*** | ***Nr3c1*** | ***Nr3c2*** | ***Nr1d1*** |
| --- | --- | --- | --- | --- | --- | --- | --- | --- | --- |
| **Prefrontal cortex** |  |  |  |  |  |  |  |  |  |
| Time in open arms in plus-maze | **r** | 0.2778 | 0.0803 | -0.0216 | 0.1871 | 0.0365 | 0.1054 | -0.0132 | -0.1749 |
|  | **p** | 0.179 | 0.703 | 0.918 | 0.371 | 0.862 | 0.616 | 0.950 | 0.403 |
| Time in contact with partner | **r** | 0.2356 | 0.4299 | 0.298 | 0.1889 | 0.4643 | 0.4332 | 0.4475 | 0.1651 |
|  | **p** | 0.257 | 0.032 | 0.148 | 0.366 | 0.019 | 0.031 | 0.025 | 0.430 |
| **Dorsal hippocampus** |  |  |  |  |  |  |  |  |  |
| Time in open arms in plus-maze | **r** | 0.2944 | -0.1058 | 0.2757 | 0.2236 | 0.044 | 0.1671 | 0.3033 | 0.1473 |
|  | **p** | 0.173 | 0.631 | 0.203 | 0.305 | 0.842 | 0.446 | 0.159 | 0.502 |
| Time in contact with partner | **r** | -0.0196 | 0.3283 | -0.0241 | 0.4556 | 0.2826 | 0.1961 | -0.0394 | 0.3224 |
|  | **p** | 0.929 | 0.126 | 0.913 | 0.029 | 0.191 | 0.370 | 0.858 | 0.134 |

**r**: Pearson’s correlation coefficient, **p**: p value.

**Figure S1.** Correlation between the relative mRNA level of genes and serum 17β estradiol concentration

1. Prefrontal cortex

r=--0.09

p=0.67

r=-0.20

p=0.36

r=-0.09

p=0.67

r=-0.09

p=0.67

r=-0.03

p=0.88

r=0.04

p=0.85

r=-0.45

p=0.03

r=-0.31

p=0.14

r=0.37

p=0.07

r=0.06

p=0.77

The X-axis represents the log (relative level of mRNA), and the Y-axis represents the log (serum level of 17β estradiol). R is the correlation coefficient, and P is the p-value of the correlation.

1. Dorsal hippocampus

r=-0.20

p=0.35

r=-0.22

p=0.34

r=0.22

p=0.33

r=0.05

p=0.83

r=0.32

p=0.15

r=0.10

p=0.65

r=0.28

p=0.20

r=0.16

p=0.47

The X-axis represents the log(relative level of mRNA), and the Y-axis represents the log(serum level of 17β estradiol). R is the correlation coefficient, and P is the p value of the correlation.
